# Promising Approaches in Plant-Based Therapies for Thyroid Cancer: An Overview of In Vitro, In Vivo, and Clinical Trial Studies

**DOI:** 10.3390/ijms25084463

**Published:** 2024-04-18

**Authors:** Ilona Kaczmarzyk, Martyna Nowak-Perlak, Marta Woźniak

**Affiliations:** Department of Clinical and Experimental Pathology, Division of General and Experimental Pathology, Wroclaw Medical University, 50-368 Wroclaw, Poland; ilona.kaczmarzyk@umw.edu.pl (I.K.); martyna.nowak-perlak@umw.edu.pl (M.N.-P.)

**Keywords:** photodynamic therapy, phytochemicals, thyroid cancer, oxidative stress, natural substances

## Abstract

Thyroid cancer, particularly undifferentiated tumors, poses a significant challenge due to its limited response to standard therapies. The incidence of thyroid cancer, predominantly differentiated carcinomas, is on the rise globally. Anaplastic thyroid carcinoma (ATC), though rare, is highly aggressive and challenging to treat. Therefore, this study aimed to collect data and explore alternative treatments, focusing on the efficacy of photodynamic therapy (PDT) combined with natural compounds as well as the potential role of phytochemicals, including quercetin, kaempferol, apigenin, genistein, daidzein, naringenin, hesperitin, anthocyanidins, epigallocatechin gallate (EGCG), resveratrol, ellagic acid, ferulic acid, caffeic acid, curcumin, saponins, ursolic acid, indole-3-carbinol (I3C), capsaicin, and piperine in thyroid cancer treatment. PDT, utilizing sensitizers activated by tumor-directed light, demonstrates promising specificity compared to traditional treatments. Combining PDT with natural photosensitizers, such as hypericin and genistein, enhances cytotoxicity against thyroid carcinoma cells. This literature review summarizes the current knowledge on phytochemicals and their anti-proliferative effects in in vitro and in vivo studies, emphasizing their effectiveness and mechanism of action as a novel therapeutic approach for thyroid cancers, especially those refractory to standard treatments.

## 1. Introduction

According to the most recent Global Cancer Observatory data from 2020, thyroid cancer is to blame for 586,000 cancer incidences globally [1]. In spite of similar death rates (0.5 per 100,000 population), thyroid cancer incidence rates are three times greater in women than in men (21 vs. 7 per 100,000 population). Certain passive tumors, such as the encapsulated follicular variety of papillary thyroid carcinoma, which account for around 20% of thyroid cancer diagnoses in the United States, have a recurrence rate of less than 1% at 15 years. Such effects occur after restricted surgery (thyroid lobectomy) [2]. All around the globe, the incidence is increasing. Nearly all thyroid cancers (>90%) are differentiated thyroid carcinomas (DTCs), which including papillary (PTC) and follicular carcinomas [3]. Undifferentiated thyroid cancers like anaplastic thyroid carcinoma (ATC) are a rare type of cancer in the US, accounting for 1.3–9.8% of the worldwide incidence [4]. Made mostly of undifferentiated follicular thyroid cells, anaplastic thyroid carcinoma is a very aggressive thyroid cancer. However, it is characterized by a very high number of mutations, especially in the p53 gene (55%), BRAF (26%), and RAS (22%), as well as genetic abnormalities, making them very difficult to treat.

Just 1.7% of thyroid tumors in the USA are anaplastic thyroid carcinomas (ATCs), making them the least prevalent kind. The majority of malignancies have a single RET mutation [5]. The most common genetic fusion is RET, particularly RET/PTC1 and RET/PTC3; fusions involving NTRK, ALK, and BRAF are uncommon. More mutations appear when the tumors differentiate, and ATCs frequently have several genetic disorders. Another important diagnostic issue is medullary thyroid cancer (MTC). MTC is a type of neuroendocrine tumor that originates from parafollicular cells, also known as “C cells”, which are derived from the neural crest and release calcitonin. As a component of multiple endocrine neoplasia (MEN), medullary thyroid cancer can develop independently in 75% of cases or naturally in 25% of cases [3].

**Figure 1:**
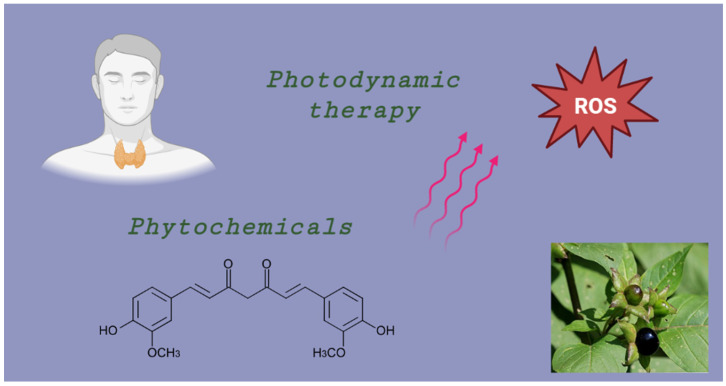
Schematic presentation of the application of thyroid treatment therapies. A showcase of the reactive oxygen species (ROS) used in PDT.

**Figure 2:**
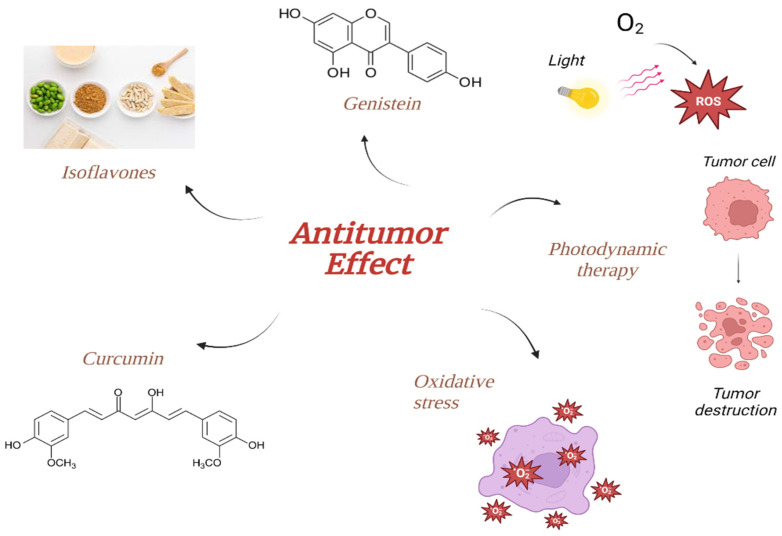
The scheme shows the antitumor effects of phytochemicals and photodynamic therapy for thyroid tumor destruction.

Despite the fact that there are a number of therapeutic alternatives, they are restricted to surgery and 131I radiation therapy, both of which have serious side effects and cannot effectively cure anaplastic thyroid carcinomas with a high degree of malignancy. In contrast to traditional chemotherapy or radiotherapy, photodynamic treatment (PDT), an antitumor technique based on sensitizer photoexcitation by tumor-directed light, has the benefit of being site-specific [6]. Some natural photosensitizers, including hypericin, are being studied as potential photosensitizers (Figure 1). The use of various substances, especially natural ones, in combination with photodynamic therapy appears to be very encouraging in terms of treating low-differentiated thyroid cancers, for which the main treatment route is usually thyroidectomy or radioactive treatment. Hypericin, for example, when paired with PDT, can effectively destroy thyroid carcinoma cells by causing intracellular reactive oxygen species (ROS) generation and the destruction of mitochondria [7]. According to the literature, natural plant compounds like phytochemicals, as well as their extracts, can be effective in treating many diseases, including those of the thyroid. Furthermore, PDT and phytochemicals have the ability to cause thyroid cancer cells to undergo apoptosis on their own, but when used together, they significantly increase these effects (Figure 2). Moreover, dietary phytochemicals have a potential to influence immune function and have a variety of pharmacological effects, such as anti-inflammatory, anticancer, and antioxidant benefits [8].

The main aim of this comprehensive literature review explores the combination of PDT with natural substances. We focused on describing in vitro and in vivo research and clinical trials evaluating natural photosensitizers to demonstrate the efficacy of alternative therapies based on natural compounds for thyroid cancers, especially low-differentiated thyroid tumors, particularly addressing patients who do not respond to standard chemotherapy or radiotherapy. Our study conducted a thorough examination of the data published in the context of phytochemicals and their usage for thyroid cancers.

## 2. The Effectiveness of PDT in the Treatment of Thyroid Cancer

Photodynamic therapy (PDT) is currently one of the most prosperous and encouraging treatments for diseases, including cancer [9]. It is based on the photoexcitation of a sensitizer (PS), which transfers energy to tissue oxygen, under the influence of laser light, producing cytotoxic reactive oxygen species (ROS) that kill cancer cells. It thereby shows significant specificity compared to, for example, radiotherapy, which is most commonly used, especially for medullary (RTC) or anaplastic (ATC) cancer, but does not necessarily carry beneficial effects for the patient. Focusing on ROS, and more specifically on actions that promote their increase, can provide a rapid pathway for inducing the apoptosis of tumor cells while effectively destroying them.

In tumor cells, glutathione (GSH) is responsible for removing reactive oxygen species, and GSH levels are very high in those cells, hindering the effect of PDT therapy, which is based specifically on the ROS-mediated destruction of cancer. Yao W. et al. [9] have shown in their analysis that it is possible to overcome these limitations. With help comes the use of nitric oxide. For many years, nitric oxide was considered a molecule that exhibited toxic effects. Although NO, along with the synthases that generate it (NOS), is believed to be involved in many thyroid diseases, earlier this century, a number of studies led to a juncture where NO was recognized as a molecule that could potentially be used for a new treatment of thyroid cancer. The research by Jiang et al. [10] further explored the effects of nitric oxide and found it to be an immunogenic inducer of cell death (ICD), triggering a specific antitumor response. The expression of NOS oxidative synthases allows for the maintenance of high levels of NO, resulting in the inhibition of thyroid tumor proliferation. Moreover, Yao W. et al., using a nanoplatform, nano-proline NO, depleted antioxidant GSH and alleviated hypoxia in tumors [9]. The MTT test revealed that the use of PDT laser radiation resulted in significant cytotoxicity in an in vitro study, with an initial cell death of <20% of cells, using treatment without PDT. Therefore, maintaining high NO levels can result in very positive effects of photodynamic therapy.

### Natural Substances and PDT

One of the primary flavonoid components of soybeans is genistein. The most abundant source of it is soybeans. Genistein has been repeatedly studied for the treatment of various cancers, and it has been shown that when combined with photodynamic therapy, it can increase its success rate. Such a combination was created against human anaplastic thyroid cancer SNU 80 cells in a study by Ahn J. et al. [11] to improve treatment efficacy. Using confocal microscopy, the production of reactive oxygen species (ROS) and membrane potential were studied. Cells were stained with rhodamine 123 and observed by fluorescence microscopy. The results showed a statistical concordance of *p* < 0.001. Also, the results of observing ROS generation showed a statistical concordance of *p* < 0.001. Genistein combined with PDT evidently enhanced the apoptotic effect of SNU 80 cells along with the inhibition of proliferation. ROS generation was also greatly enhanced.

The follow-up conjunction with photodynamic therapy, reviewed by Saswata Ch. et al. [12] of the Beckman Laser Institute Korea, is based on the use of sulforaphene, a natural isothiocyanate from brassica vegetables that exhibits broad anticancer activity, especially the most aggressive ones like anaplastic thyroid cancer (ATC). Employing photofrin PDT together with sulforaphene, a significant decrease in tumor FRO cell proliferation and apoptosis can be observed due to the production of large amounts of reactive oxygen species and membrane depolarization. Thanks to the relevant equations created by the researchers, the conjugation of the therapy with the analyzed substance was evaluated. Using the DRI (dose reduction index), the decrease in toxicity was checked by lowering the drug dose without affecting the efficacy. As it turned out, the combination group of two or more drugs shows strong synergism and the toxicity of the combined treatment decreases. The authors performed a statistical analysis of the samples, which showed *p* < 0.05, *p* < 0.01, and *p* < 0.001, respectively. The viability of FRO cells at a photofrin concentration of 1.5 μg/mL decreased rapidly to 50.5 ± 4.37%, and at 6.25 μg/mL, the value was 20.5 ± 0.3728%. This is further evidence that combining natural substances with PDT has positive therapeutic effects. Another natural photosensitizer is hypericin (HYP), isolated from St. John’s wort, which is very often used against hard-to-treat, low-differentiated cancers. In an in vivo study in mice, Hyejin Kim et al. [13] investigated the behavior of anaplastic thyroid carcinoma (ATC) cells, after HYP injection and laser use, in terms of ROS production and the presence of membrane potential and mitochondrial destruction. As a result of the production of high amounts of these factors, more rapid cell death occurred and the tumor was completely eradicated. The results of a statistical analysis showed statistical correlation, with *p* < 0.05. All of the effects of the treatment indicate the wide possibility of using photodynamic therapy in combination with specific substances, especially the best current prognostic substances extracted from plants.

## 3. Phytochemicals in Thyroid Cancer Treatment

The treatment of thyroid malignancies is still a challenge; therefore, based on the literature, phytochemicals provide effective anticancer therapy. As a result of their anticarcinogenic qualities, they have been linked to favorable effects in epidemiologic and experimental research. Their leading role in killing cancer cells is based on the generation of reactive oxygen species (ROS). ROS play a part in inflammation and a number of ongoing illnesses [14]. ROS production in cancer cells is one of the mechanisms underlying the synergistic cytotoxicity of combination therapies. The accumulation of unfolded or misfolded proteins in response to oxidative stress causes ER stress, allowing amplification loops that may aid in the transition from adaptive to fatal unfolded protein response (UPR) [15]. The primary source of cellular ROS is the mitochondria, making the stimulation of mitochondrial ROS production one of the main anticancer strategies [16]. Accordingly, it is suggested that the level of ROS production and the effectiveness of its dynamic regulation may decide how cancer cells respond to chemotherapy.

The most prevalent class of natural antioxidants is phenolic compounds, which have both direct and indirect antioxidant action that lowers oxidative stress [17]. Biological processes, such as antioxidative action, may be modified by polyphenols in thyroid cancer. It might be crucial to target enzymatic ROS sources without influencing the body’s redox state. Unchecked chronic inflammation produces potentially dangerous oxygen species continuously, which can lead to DNA damage, genomic alterations, and tumor growth. The production of proangiogenic growth factors like cytokines and VEGF as well as inflammatory molecules like IFN, TNF, and IL-1/IL-6 is also unending.

Though anaplastic thyroid cancer (ATC) accounts for less than 2% of thyroid tumors (TCs), it causes about 50% of deaths [18]. The curative effectiveness of treatments is unsatisfactory and 40–60% of ATC patients pass away within a few months of their diagnosis. Numerous health advantages of resveratrol, 3,5,4′-trihydroxystilbene, include its anti-inflammatory, antioxidant, and cancer-preventive properties [19]. Results from Xu Zheng et al. [20] show that resveratrol increases ROS production and oxidative-related cellular lesions in resveratrol-sensitive THJ-16T cells by triggering the ROS–mitochondrial signal pathway. By using the MTT test, the effects of resveratrol on the proliferation of two ATC cells were clarified. Resveratrol inhibited the proliferation of THJ-16T cells in a time-dependent manner, according to the results of the MTT assay (12 h, *p* < 0.05; 24 h and 48 h, *p* < 0.01). It is known that resveratrol inhibits the growth of cancer cells while having no negative effects on the equivalent normal cells. The results above show that resveratrol’s suppressive effects on ATC cells are closely linked to increased ROS generation and oxidative damage.

In this article, the most promising natural substances are discussed. Additional potential treatments are being researched. Further compounds include thiols and nutraceuticals such as piperine. Indole-3-carbinol (I3C) may be found in cruciferous vegetables. I3C and its acid-catalyzed dimer, 3,3′-diindolylmethane (DIM), have been studied for their antitumor activity (TCa) by Raj K. Tiwari et al. [21]. Primary human goiter cells as well as four distinct cell lines representing follicular thyroid carcinoma (CGTH-W-1 and ML-1) and papillary thyroid carcinoma (B-CPAP and 8505-C) were used in the investigation. Using the XTT assay, cell survival and IC50 values for I3C and DIM were determined, and flow cytometry was used to analyze the cell cycle distribution. The results showed that I3C together with DIM has anti-proliferative effects in both papillary and follicular thyroid cancer. DIM also inhibited the growth of primary goiter cells by 70% compared to untreated controls. Piperine is a nutraceutical that may have an impact on cell cycle regulators and the genetic expression of cell markers. Using the matching chemical compounds, Esposito et al. [22] showed the inhibitory effect on cancer cell proliferation. Curcumin extract, piperine, and vitamin E, either singly or in combination, were dissolved in culture medium and applied to TPC-1 papillary thyroid cancer cells for a duration of 48 h. Consequently, it was demonstrated that the treatment may have an impact on apoptosis activators or inhibitors (BAX, procaspase3, Bcl-2) as well as cell cycle regulators (cyclin D1, β-catenin, p21, p53). However, more research is still needed to confirm the effectiveness of these compounds.

One very promising new therapeutic is polyphenols. Fruits and vegetables contain polyphenolic phytochemicals (PPs) that prevent cancer in vivo and in vitro. Apigenin has demonstrated antitumor actions in BCPAP cells of a papillary thyroid cancer (PTC) cell line in a study by Li Zhang et al. [23] The TUNEL test revealed that apigenin increased the generation of reactive oxygen species (ROS), which in turn caused a considerable amount of DNA damage. Hesperetin, a flavonoid, decreases ATC cell growth, primarily by apoptosis, and promotes the Notch1 signaling cascade. The effects of this substance were assessed in a study by Greenman Y. et al. [24] using the anaplastic thyroid carcinoma (ATC) cell line HTh7. Cell proliferation and the activating role of Notch1 were evaluated. Hesperetin-treated ATC cells showed increased expression of Notch1, its downstream effector hairy and enhancer of split 1 (Hes1), as well as Hes1 coupled to the YRPW motif. Francesca De Amicis et al. [25] conducted an analysis that revealed the impact of epigallocatechin gallate (EGCG) on the motility and proliferation of human follicular carcinoma (WRO) and papillary thyroid cancer (FB-2) cell lines. The findings demonstrate that the growth of FB-2 and WRO cells was dose-dependently decreased by EGCG administration (10, 40, and 60 µM). Xianglong Meng et al. [26] showed that ellagic acid (EA) may be a promising medication option for the treatment of anaplastic thyroid carcinoma (ATC) since it suppresses the growth, migration, and invasion of ATC cells. The ATC cell line BHT-101 and the normal human thyroid cell line Nthy-ori3-1 were employed, and the Cell Counting Kit 8 (CCK-8) was used to conduct a cytotoxicity assay. The PI3K/Akt pathway was evaluated by measuring and computing the levels of Akt protein phosphorylation. EA therapy inhibited the Wnt/β-catenin and PI3K/Akt pathways. The use of caffeic acids in treatment can also have promising results. R Zamora-Ros et al. [27] assessed the effects of caffeic acid and its dehydrogenated metabolite on differentiated thyroid cancer cells using multivariable-adjusted conditional logistic regression models. They examined how acid and its dehydrogenated metabolite affected thyroid cancer cells that had undergone differentiation. Papillary TCs had comparable outcomes. The study suggests that a decreased risk of papillary TC may be linked to high blood levels of caffeic acid. Examples of research include quercetin, resveratrol, and genistein and kaempferol. Hee Joon Kang et al.’s [28] examination of these compounds showed that cell growth was inhibited by genistein, resveratrol, quercetin, and kaempferol in a dose-dependent manner. Using a mouse model of F9 embryonal carcinoma cells, this study attempted to show that specific phytochemicals have the ability to induce anti-proliferation and redifferentiation in thyroid cancer cell lines. MTT tests were conducted after each agent was administered for 24, 48, 72, 96, and 120 h. These substances inhibited cell growth for up to 120 h (*p* < 0.05). Genestein, resveratrol, and quercetin have the ability to suppress CD97, a typical marker of differentiation in thyroid carcinoma that is induced by EGF, as demonstrated in the above analyses.

Resveratrol is found naturally in grapes, berries, and a number of therapeutic plants. Since its chemopreventive and anticancer effects were originally discovered in the late 1990s, they have gained widespread recognition. It was previously believed that Notch1 is a key signaling pathway that controls gene expression in the thyroid and determines the loss of thyroid cellular components. The results of the study carried out by Xiao-Min Yu et al. [29] demonstrated that resveratrol significantly slows the growth of ATC cells, inducing both the arrest of cell cycle progression and apoptosis. Additionally, resveratrol increased the expression of specific thyroid genes in ATC, dependent on Notch1 activation. These findings suggest that compounds activating Notch1 may be further investigated as a potential treatment for patients with ATC. An MTT assay was used to evaluate cell viability 72 h following resveratrol treatment at various concentrations up to 400 μmol/L in order to establish the effective dose against ATC. Resveratrol significantly inhibited the development of both examined cell lines in a dose-dependent manner (*p* < 0.05).

Turmeric, a widely used food coloring and flavoring agent, contains a natural polyphenolic compound called curcumin. It has been demonstrated that curcumin possesses antiviral, antibacterial, antioxidant, anti-inflammatory, anti-proliferative, and antiangiogenic properties. Researchers from the University of Ulsan College of Medicine, Jung Min Hong et al. [30] found that concurrent curcumin treatment improved the sensitivity of ATC cells to docetaxel, and they looked at the apoptotic signaling pathways in the treated cells. The growth-inhibitory and proapoptotic actions of docetaxel were demonstrated for the first time to be enhanced by curcumin, perhaps through interference with the signaling pathways of NF-κB and cyclooxygenase-2 (COX-2). These findings supported the occurrence of docetaxel-induced NF-κB activation as well as the fact that curcumin prevented it. The expression of the COX-2 protein was also observed to be down-regulated after curcumin therapy. *p* < 0.05 indicated a statistically significant difference. By reducing the breakpoint concentration needed for docetaxel to suppress cell proliferation and induce cell death, curcumin improves the therapeutic effectiveness of docetaxel.

Naringin is a flavonoid found primarily in grapefruit and other citrus fruit. This compound has a variety of biological actions, including effects against oxidative stress and tumor prevention, according to studies. Researchers Jun Zhou et al. [31] investigated how naringin inhibits the development of AGS tumor cells by activating MAPK pathways and inhibiting the PI3K/Akt/mTOR cascade. Naringin was applied to TPC-1 and SW1736 cells for 24, 48, or 72 h at 37 °C, respectively. The results of the MTT assay, which was used to measure cell proliferation, showed that naringin decreased TPC-1 and SW1736 cell proliferation in a dose- and time-dependent manner. The results of this study showed that naringin inhibited thyroid cancer cell growth and induced cell apoptosis by controlling the expression of genes associated with cell growth and apoptosis and by activating the PI3K/AKT pathway. Based on these findings, naringin may be a useful treatment for TC.

Daidzein is a naturally occurring substance that is only present in legumes, specifically soybeans, and it is structurally an isoflavone. Researchers tested the possibility that the novel isoflavone derivative, carboxy-daidzein-tBoc (cD-tBoc), could inhibit the proliferation of the human MTC TT cell line through interaction with estrogen receptor β [32]. In vitro, in animal models, and in humans, normal thyroid C cells have been shown to secrete more calcitonin in response to estrogen stimulation, indicating the presence of estrogen receptors in these cells. By RT-PCR or immunostaining with various antibodies, ER has been found in the majority of MTC samples. The present study’s major discovery is that cD-tBoc potently reduced TT-cell proliferation by inducing both cell apoptosis and necrosis. Daidzein, the parent substance of cD-tBoc, has been demonstrated to have a higher affinity for ER. The maximum effect of cD-tBoc on apoptosis (1350–1750% increase in histone–DNA fragments) was already present at the lowest concentration examined (0.0312 μM; *p* < 0.001). The findings suggest that this property may be used in the creation of strong anticancer medications for the treatment of medullary thyroid carcinoma.

Fruits and vegetables such as blueberries, cranberries, pears, cherries, apples, grapefruits, lemons, peaches, potatoes, lettuce, and spinach are rich in ferulic acid (FA), a frequent dietary plant phenolic component. Researchers Yavuz Dodurga et al. [33] examined how FA affected the human TT medullary thyroid carcinoma cell line’s ability to invade, transmit cell cycle signals, and express genes involved in apoptosis pathways. A novel gene called URG4/URGCP, involved in cell cycle regulation, is potentially a molecular target for the treatment of this type of cancer. According to the study’s findings, FA was able to lower URG4/URGCP gene expression, leading to a reduction in cell invasion, cell migration, and colony formation. Compared to control cells, the URG4/URGCP mRNA expression in FA-treated cells was considerably reduced (*p* < 0.05). FA may be an innovative therapy option for thyroid cancer, particularly medullary thyroid carcinoma.

**Figure 3:**
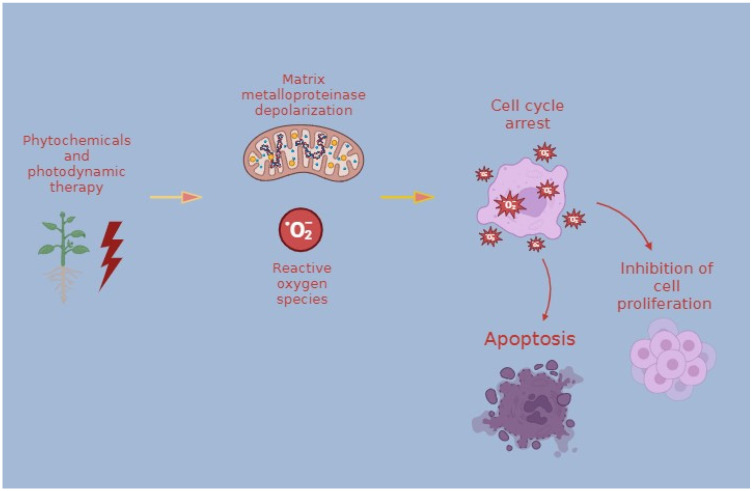
Proapoptotic effects of phytochemicals and photodynamic therapy mechanism. Natural photosensitizers, when used alone or in combination with PDT, induce cell cycle arrest, inhibition of proliferation, and cell death by apoptosis and necrosis, as well as leading to increased oxidative stress, altered cancer cell death signaling pathways, increased cytotoxicity, and DNA damage in thyroid cancer cells.

The next compounds are terpenoids. Ursolic acid (UA) has anti-proliferative and differentiation effects on human cancer cell lines, according to Irene Bonaccorsi et al.’s [34] investigation of the drug’s effects on ARO anaplastic thyroid cancer cells. Cells were incubated with 15 µM of UA for three 96 h cycles. Consequently, a consistent inhibition of cell proliferation was observed. Furthermore, endogenous reverse transcriptase (RT) activity in cancer cells is inhibited by UA, and this has lately emerged as a critical target for cancer differentiation therapy. One of the main triterpenoid saponins obtained from Bupleurum falcatum L is saikosaponin-d, which is frequently given as a treatment for infectious and inflammatory disorders in China, Japan, and other Asian nations. Researchers Liu et al. [35] examined the effect of saikosaponin-d on human undifferentiated thyroid cancer’s proliferative and apoptotic processes by the use of the ARO anaplastic thyroid carcinoma cell line. The MTT experiment demonstrated that saikosaponin-d therapy effectively and dose- and time-dependently reduced the proliferation of ARO cells. These findings suggest that the anti-proliferative property of saikosaponin-d inhibited the growth of anaplastic thyroid cancer cells. The apoptosis rate of ARO was detected by flow cytometry (*p* < 0.05; *p* < 0.01). In summary, saikosaponin-d may be a brand-new drug that could be used in the future to treat anaplastic thyroid tumors (Figure 3).

The last important group of compounds in this paper are alkaloids. One of them is capsaicin. The major compound in red or spicy chili peppers of the genus Capsicum, known as capsaicin, is primarily in charge of giving chili peppers their pungent flavor. Since ancient times, people have used Capsicum annuum extensively as a spicy flavoring in order to stimulate their appetite. It is also occasionally used to alleviate symptoms of the common cold. Because of its anticancer properties, capsaicin has recently gained more and more attention. According to a study by Shichen Xu et al. [36], capsaicin can directly activate TRPV1 (transient receptor potential vanilloid type 1) receptors in BCPAP cells, which prevents the EMT (epithelial–mesenchymal transition) and, as a result, prevents cell adhesion, migration, and invasion. In a dose-dependent manner, capsaicin prevented BCPAP cells from migrating. Applying an unpaired Student’s t test, a statistical analysis was carried out. Following a 48 h treatment with 100 μM of capsaicin as opposed to a solvent control, the wound closure rate dropped from 90.26 ± 5.35% to 18.04 ± 2.71% (*p* < 0.01). The findings indicate that capsaicin at least partially suppresses EMT to prevent thyroid cancer cell metastasis. A new approach to treating papillary thyroid cancer, particularly metastasis, may be developed with the use of capsaicin therapy as an innovative target.

## 4. Discussion

The potential anticancer effects of phytochemicals on thyroid cancer development are currently the focus of numerous scientific studies (Table 1). In a study conducted by Kang et al. [28], it was found that polyphenol phytochemicals, such as genistein, resveratrol, quercetin, and kaempferol, can suppress thyroid cancer cell proliferation and stimulate redifferentiation, indicating their potential use in thyroid cancer redifferentiation treatment. Furthermore, indole-3-carbinol shows its anti-proliferative properties as a thiol molecule [21], and curcumin has an effect on papillary, anaplastic, and medullary thyroid cancer cell viability and invasiveness [22,30,37]. A study conducted by Meng et al. [26] revealed that ellagic acid inhibits the cell growth, migration, and invasion of anaplastic thyroid cancer cells. Additionally, other research demonstrates its antitumor, antimetastatic, and antiangiogenic activities in various types of cancer, including colorectal, breast, prostate, lung, and melanoma cancers [38]. Among terpenoids, a phytochemical known as ursolic acid was found by Cao et al. [39] to effectively suppress the growth, migration, and invasion of human papillary thyroid carcinoma cells. Esposito et al. [22] discovered that curcumin, in combination with piperine and vitamin E, can decrease the viability of papillary thyroid carcinoma cells. Studies conducted on piperine alone demonstrated a widespread inhibitory effect on cell proliferation across various cancers, including breast, prostate, osteosarcoma, colon, and melanoma [40,41,42]. On the contrary, Xu et al. [36] uncovered that another phytochemical, capsaicin, has the potential to inhibit various stages of metastasis without impacting the viability of papillary thyroid cancer cells. In anaplastic thyroid cancer cells, mitochondrial calcium accumulation occurs, leading to apoptosis [43]. Another polyphenol molecule, daidzein, was found to inhibit thyroid cancer cell proliferation by apoptosis rather than necrosis in a dose-dependent manner [44]. Furthermore, research conducted by Zhou et al. [31] demonstrated that naringenin enhances anticancer effects in thyroid cancer, not only by diminishing cell proliferation but also by inducing apoptosis through an alternative mechanism, specifically the inhibition of the PI3K/AKT signaling pathway. Additionally, numerous phytochemical compounds have been recognized for their anticancer properties, including ferulic acid. Dodurga et al. [33] identified that ferulic acid inhibits growth and invasiveness in TT medullary thyroid cancer cells, primarily by inducing cell cycle arrest and promoting apoptosis. Similarly, caffeic acid has been found to exhibit comparable effects [27], while hesperetin has been shown to induce apoptosis and facilitate cell differentiation in anaplastic thyroid cancer [45]. In a similar manner, saponins have been identified by Liu et al. [35] to play a pivotal role in the induction of apoptosis and cell cycle arrest. Furthermore, epigallocatechin gallate, as a phytochemical, can lead to apoptosis/cell death pathways and influence the genes associated with epigenetic mechanisms [46], but also inhibits epithelial-to-mesenchymal transition [25]. Contrary to the previously mentioned compounds that induce cell death via apoptosis, Cheng et al. showed that punicalagin promotes apoptosis-independent autophagic cell death in human papillary thyroid carcinoma cells [47]. Furthermore, a study conducted by Zhang et al. [23] demonstrated that apigenin treatment induces cell death in human papillary thyroid carcinoma cells. Nonetheless, this is not the only physiological effect in anticancer treatment; it may also promote apoptosis, necroptosis, and ferroptosis, with the specific response varying by cancer type [48].

Both in vitro and in vivo experimental research studies suggest their significant potential in mitigating the development and progression of thyroid cancer. Furthermore, the presented results indicate that phytochemicals can exert anticancer effects through two mechanisms: Firstly, by reducing the risk of cancer development through the inhibition of early carcinogenesis stages, such as initiation, promotion, the modulation of cell cycle arrest, the induction of apoptosis, and decreasing invasion, migration, and colony formation. Secondly, when incorporated into photodynamic therapy, they enhance cancer cell sensitivity to treatment, potentially limiting the progression of thyroid cancer. Furthermore, as plants serve as a rich source of numerous phytochemicals, it is often challenging to distinctly attribute significant anticancer effects to specific chemical compounds in clinical studies. Nevertheless, evidence suggests that phytochemicals and photodynamic therapy may have therapeutic potential for thyroid cancers. Therefore, more experiments are needed to evaluate detailed strategies to use them in clinics.

## Figures and Tables

**Table 1:** Table presents summary of the literature data in regard to phytochemicals in thyroid cancer treatment in vitro, in vivo, and in clinical trials.

Phytochemicals	Classification			Natural Occurrence	Effects	Application with References		
	Group	Subgroup	Class			Papillary Thyroid Cancer	Anaplastic Thyroid Cancer	Medullary Thyroid Cancer
Quercetin	Polyphenols	Flavonoids	Flavonols	Onion, kale, red wine, berries, buckwheat, red grapes, tea, apples	Inhibition of growth of cancer cells; induces down-regulation	[49,50]	[28]	[50]
2. Kaempferol	Polyphenols	Flavonoids	Flavonols		Inhibits cell growth in a dose-dependent manner	[51]	[28]	ND
3. Apigenin	Polyphenols	Flavonoids	Flavones	Celery, herbs, parsley, chamomile, rooibos tea, capsicum pepper	Inhibit cancer cell, induces autophagic cell death	[23]	[52]	ND
4. Genistein	Polyphenols	Flavonoids	Isoflavones	Soya, beans, chickpeas, alfalfa, peanuts	Inhibits tumor growth and improves the response to conventional therapy	ND	[28]	[53]
5. Daidzein	Polyphenols	Flavonoids	Isoflavones		Retards the growth of human thyroid carcinoma cell lines	[32]	ND	[24]
6. Naringenin	Polyphenols	Flavonoids	Flavanones	Citrus fruit	Induces cell apoptosis	[31,45]	ND	ND
7. Hesperetin	Polyphenols	Flavonoids	Flavanones		Induces cellular differentiation	ND	[45]	ND
8. Anthocyanidins	Polyphenols	Flavonoids	-	Red grapes, pomegranates, blueberries, cherries, strawberries, blackberries, raspberries	Promotes cell death	[54]	ND	ND
9. Epigallocatechin gallate (EGCG)	Polyphenols	Flavonoids	Flavan–3–ols tannins	Tea, chocolate, grapes	Inhibits growth and epithelial-to-mesenchymal transition	[25]	[46]	ND
10. Resveratrol	Polyphenols	Flavonoids	Flavanolols	Grapes, berries, peanuts, blueberries, raspberries, wine	Inhibits tumor growth	[55,56]	[28,29]	[57]
11. Ellagic acid	Polyphenols	Phenolic acids	Hydrobenzoic acids	Blackberries, grape seed, pomegranates, raspberries, tea, vanilla	Inhibits cell proliferation, migration, and invasion	ND	[26]	ND
12. Ferulic acid	Polyphenols	Phenolic acids	Hydroxycinnamic acids	Blueberries, cinnamon, coffee, kiwi fruits, plums, wheat bran	Effects the cell cycle, apoptosis, invasion, and colony formation	ND	ND	[33]
13. Caffeic acid	Polyphenols	Phenolic acids	Hydroxycinnamic acids		Modulates cell cycle arrest, apoptosis, invasion, migration, and colony formation	[27]	ND	ND
14. Curcumin	Polyphenols	Non-flavonoid polyphenols	Curcuminoids	Turmeric	Effects the viability, migration, and invasion of cancer cells	[22,37]	[30]	[30]
15. Saponins	Terpenoids	Non-carotenoid terpenoids	-	Chickpeas, soya beans, coffee, tea	Induction of apoptosis and cell cycle arrest	ND	[35]	ND
16. Ursolic acid	Terpenoids	Non-carotenoid terpenoids	-	Apples, cranberries, peppermint, prunes, oregano, thyme	Inhibits tumor cell proliferation	ND	[34]	ND
17. Indole–3–carbinol (I3C)	Thiols	-	Indoles	Broccoli, brussels sprouts	Anti-proliferative effects	[21]	ND	ND
18. Capsaicin	Alkaloids	-	-	Chili	Inhibits the metastasis of cancer	[36]	[43]	ND
19. Piperine	Others	-	-	Black peppers	Inhibitory effect on cell proliferation	[22]	ND	ND

ND—no data.

## Data Availability

All data are available in the manuscript.
